# Data on the synthesis and mechanical characterization of polysiloxane-based urea-elastomers prepared from amino-terminated polydimethylsiloxanes and polydimethyl-methyl-phenyl-siloxane-copolymers

**DOI:** 10.1016/j.dib.2018.04.083

**Published:** 2018-04-30

**Authors:** Natascha Riehle, Tobias Götz, Andreas Kandelbauer, Günter E.M. Tovar, Günter Lorenz

**Affiliations:** a Reutlingen Research Institute, Reutlingen University, Alteburgstr. 150, 72762 Reutlingen, Germany; b School of Applied Chemistry, Reutlingen University, Alteburgstr. 150, 72762 Reutlingen, Germany; c Institute of Interfacial Process Engineering and Plasma Technology IGVP, University of Stuttgart, Nobelstr. 12, 70569 Stuttgart, Germany; d Fraunhofer-Institute for Interfacial Engineering and Biotechnology IGB, Nobelstr. 12, 70569 Stuttgart, Germany

**Keywords:** Amino-terminated polydimethylsiloxanes, Polysiloxane-based urea elastomers, ^1^H and ^29^Si NMR spectroscopy, FTIR-ATR spectroscopy, Tensile properties, Mechanical hysteresis

## Abstract

This article contains data on the synthesis and mechanical characterization of polysiloxane-based urea-elastomers (PSUs) and is related to the research article entitled “Influence of PDMS molecular weight on transparency and mechanical properties of soft polysiloxane-urea-elastomers for intraocular lens application” (Riehle et al., 2018) [1]. These elastomers were prepared by a two-step polyaddition using the aliphatic diisocyanate 4,4′-Methylenbis(cyclohexylisocyanate) (H_12_MDI), a siloxane-based chain extender 1,3-Bis(3-aminopropyl)-1,1,3,3-tetramethyldisiloxane (APTMDS) and amino-terminated polydimethylsiloxanes (PDMS) or polydimethyl-methyl-phenyl-siloxane-copolymers (PDMS-Me,Ph), respectively. (More details about the synthesis procedure and the reaction scheme can be found in the related research article (Riehle et al., 2018) [1]).

Amino-terminated polydimethylsiloxanes with varying molecular weights and PDMS-Me,Ph-copolymers were prepared prior by a base-catalyzed ring-chain equilibration of a cyclic siloxane and the endblocker APTMDS. This DiB article contains a procedure for the synthesis of the base catalyst tetramethylammonium-3-aminopropyl-dimethylsilanolate and a generic synthesis procedure for the preparation of a PDMS having a targeted number average molecular weight ${\bar{M}}_{n}$ of 3000 g mol^−1^. Molecular weights and the amount of methyl-phenyl-siloxane within the polysiloxane-copolymers were determined by ^1^H NMR and ^29^Si NMR spectroscopy. The corresponding NMR spectra and data are described in this article.

Additionally, this DiB article contains processed data on *in line* and *off line* FTIR-ATR spectroscopy, which was used to follow the reaction progress of the polyaddition by showing the conversion of the diisocyanate. All relevant IR band assignments of a polydimethylsiloxane-urea spectrum are described in this article.

Finally, data on the tensile properties and the mechanical hysteresis-behaviour at 100% elongation of PDMS-based polyurea-elastomers are shown in dependence to the PDMS molecular weight.

**Specifications table**

**Table:** 

Subject area	*Chemistry*
More specific subject area	*Macromolecular Chemistry, Polymer Chemistry, Siloxane-based Elastomers*
Type of data	*Tables, figures, text file*
How data was acquired	^*1*^*H NMR and*^*29*^*Si NMR spectroscopy (Avance III 300 MHz, Bruker Optik GmbH, Ettlingen, Germany); titration of amino-end groups; Size Exclusion Chromatography (SEC) (1260 Infinity II GPC-SEC Analysis System from Agilent Technologies Deutschland GmbH, Waldbronn, Germany) equipped with 3 PSS SDS columns (PSS Polymer Standards Service) and a refractive index detector; FTIR-ATR-spectroscopy (off line: Perkin Elmer FTIR spectrometer (Frontier®)) with ZnSe-Diamond ATR unit (Perkin Elmer Germany GmbH, Rodgau, Germany) / in line: Mettler Toledo ReactIR 45 m® ATR-FTIR spectrometer equipped with a SiComp (Silicon) probe (Mettler Toledo GmbH, Gießen, Germany), tensile tests were performed with a Zwick model 81565 using a 100 N load cell (Zwick GmbH & Co. KG, Ulm, Germany)*
Data format	*Raw, analyzed and processed data*
Experimental factors	*NMR spectroscopy: samples were filtrated prior to measurement.*
	*SEC: samples were filtrated prior to analysis.*
	*off line FTIR spectroscopy: samples (dissolved in THF) at different stages of the polyaddition were taken from the reaction vessel and placed on the ATR crystal. After evaporation of the solvent in a continuous flow of nitrogen, a very thin polymer film was obtained and subsequently measured.*
	*Tensile tests: sheets were dried and annealed at* 80 °C *in a vacuum chamber for* 24 h *and left at ambient temperature for at least* 72 h *before measurements.*
Experimental features	*Analysis of molecular weights of PDMS and PDMS-Me-Ph-copolymers by different methods (end-group titration,*^*1*^*H NMR, SEC) and comparison to theoretical calculated molecular weight. Determination of incorporated amount of methyl-phenyl-siloxane within PDMS-Me-Ph-copolymers by*^*29*^*Si NMR spectroscopy. Evaluation of polyaddition reaction speed by means of in line FTIR spectroscopy. Determination of the effect of PDMS molecular weight on the mechanical properties and hysteresis behaviour of PDMS-based urea-elastomers.*
Data source location	*NMR spectra were measured at University of Tübingen, Germany.*
	*SEC measurements were performed at University of Stuttgart, Germany.*
	*FTIR spectra and mechanical testings were performed at Reutlingen University, Germany*
Data accessibility	*The data are available within this article*

**Value of the data**

• The presented data provides a simple method of preparing amino-terminated polydimethylsiloxanes and polydimethyl-methyl-phenyl-siloxane-copolymers within a broad range of molecular weights (3000 to >30,000 g mol^−1^).
• The ^1^H and ^29^Si NMR spectra can be used for characterization of the PDMS and PDMS-Me,Ph-copolymers regarding molecular weight and composition.
• The FTIR data can be used by other researchers to estimate reaction times of amino-terminated macromonomers and low molecular weight diamines towards aliphatic diisocyanates when preparing polyurea-elastomers.
• The provided data about mechanical properties of polysiloxane-urea-elastomers can be used to evaluate the effect of the molecular weight of the polysiloxane on the resulting elastomer materials.
• The data can be used by other researchers to design a soft urea-based-elastomer with predictable mechanical properties for biomedical or coating applications for instance.

## 1 Data

The presented data on synthesis of PDMS and PDMS-Me,Ph-copolymers as well as the synthesis data of the resulting polysiloxane-based urea-elastomers are raw data obtained from a single synthesis. Molar ratios, conversions and theoretical molecular weights of the polysiloxanes, displayed in Tables 1 and 2, were calculated based on the initial weights of used monomers. Spectral data from FTIR-ATR spectroscopy, shown in Fig. 4, Fig. 5, Fig. 6 were processed (*in line* spectroscopy: smoothed data using moving-average/*off line* spectroscopy: average spectra from 6 scans). Data on mechanical properties and hysteresis-behaviour of PSUs were obtained from 5 repeated measurements (tensile properties) and from 3 repeated measurements (hysteresis) and are displayed as mean value including standard deviation.

**Table 1:** Synthesis data and number average molecular weights of α,ω-Bis(3-aminopropyl)-polydimethylsiloxanes. The number of the PDMS refers to the molecular weight determined by ^1^H NMR with T standing for ‘thousand’.

**PDMS**	**Molar ratio (D**_**4**_**/APTMDS)**	**Conversion (%)**	${\bar{M}}_{n}$**Theoretical**	${\bar{M}}_{n}$**Titration**	${\bar{M}}_{n}$**NMR**
**3T**	11.1/1.0	82.12	2897	3295	3416
**6T**	22.8/1.0	83.83	5881	6108	6407
**9T**	34.8/1.0	78.61	8308	9580	9570
**12T**	46.6/1.0	85.27	12,004	12,576	12,404
**15T**	58.2/1.0	84.23	14,747	14,862	15,203
**18T**	70.0/1.0	81.79	17,179	18,308	18,443
**23T**	82.3/1.0	84.67	20,883	22,684	23,056
**26T**	96.4/1.0	86.10	24,821	25,753	26,050
**31T**	109.7/1.0	88.64	29,056	30,647	31,047
**33T**	118.2/1.0	89.03	31,431	32,502	33,191

**Table 2:** Synthesis data and number average molecular weights of α,ω-Bis(3-aminopropyl)-polydimethyl-methyl-phenyl-siloxane-copolymers. The number of the PDMS refers to the incorporated amount of methyl-phenyl-siloxane in mol%.

**PDMS**	**Molar ratio (D**_**4**_**/D**_**4**_^**Me,Ph**^**/APTMDS)**	**Methyl-phenyl siloxane (mol%)**^a^	**Conversion (%)**	${\bar{M}}_{n}$**Theoretical**	${\bar{M}}_{n}$**Titration**	${\bar{M}}_{n}$**NMR**
**Ph2**	67.3/1.4/1.0	2.01	81.96	17,170	18,370	19,583
**Ph4**	65.6/2.7/1.0	4.00	83.70	17,752	18,264	19,683
**Ph6**	63.3/4.0/1.0	6.03	84.47	17,925	20,054	19,857
**Ph8**	60.9/5.3/1.0	8.22	85.18	18,058	19,919	21,478
**Ph10**	58.6/6.5/1.0	9.95	91.88	19,479	19,334	21,405
**Ph12**	56.5/7.7/1.0	12.12	93.83	19,904	20,500	22,001
**Ph14**	54.4/8.9/1.0	13.96	93.29	19,792	19,715	19,680

a calculated from ^29^Si NMR spectra.

## 2 Experimental design, materials and methods

### 2.1 General synthesis procedures

#### 2.1.1 Synthesis of tetramethylammonium-3-aminopropyl-dimethylsilanolate catalyst

**Fig. 1:**
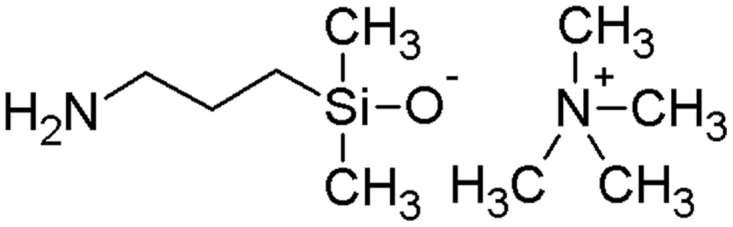
Structure of the catalyst tetramethylammonium-3-aminopropyl-dimethylsilanolate.

The synthesis of tetramethylammonium-3-aminopropyl-dimethylsilanolate (see structure in Fig. 1) as a basic catalyst for the ring-chain equilibration of cyclic and linear siloxanes was carried out according to a method described by Hoffman and Leir [2]. APTMDS (8.13 g, 33.0 mmol) and TMAH (11.88 g, 66.0 mmol) were dissolved in THF (20 mL) and the solution was added to a 100 mL three-neck round-bottom flask, equipped with a reflux condenser, a magnetic stir bar and a nitrogen in- and outlet. The reaction mixture was heated to 80 °C and stirred under reflux for 2 h under a continuous flow of nitrogen. After 2 h, the condenser was removed and THF was distilled off from the crude product under aspirator vacuum. The resulting slightly yellow product was dried under a vacuum of 0.1 mbar for 5 h at 70 °C using a Schlenk-Line. After cooling to room temperature, the crude product was resuspended in 50 mL THF and was filtered and washed 3 times with 20 mL THF under aspirator vacuum until the product became a white crystalline solid. The catalyst was dried for 3 h under a vacuum of 0.1 mbar at room temperature and stored until usage at 10 °C under nitrogen. Yield: 14.0 g; 70%.

^1^H NMR (300 MHz, DMSO-d6) δ: 3.19 (s, 12H, (CH_3_)_4_), 2.39 (t, *J*=6 Hz, 2H; CH_2_), 1.29 (q, *J*=6 Hz, 2H; CH_2_), 0.15 (m, 2H; CH_2_), −0.31 (s, 6H, (CH_3_)_2_).

#### 2.1.2 Synthesis of α,ω-Bis(3-aminopropyl)-polydimethylsiloxanes

As an example, the synthesis of α,ω-Bis(3-aminopropyl)-polydimethylsiloxane with a targeted molecular weight of 3000 g mol^−1^ was performed as follows. 30% (w/w) of the total amount of D_4_ (19.5 g, 65.7 mmol) was weighed into a 100 mL three-neck round-bottom flask, equipped with a magnetic stir bar and a nitrogen in- and outlet. APTMDS (4.922 g, 19.8 mmol) and catalyst (28 mg, 0.04% (w/w)) were added and the mixture was stirred at 80 °C for 30 min under a continuous flow of nitrogen. Then, the remaining portion of D_4_ (45.5 g, 153.4 mmol) was added dropwise via a dropping funnel over a period of about 2–3 h. The reaction mixture was equilibrated for 24 h at 80 °C. The ring-chain-equilibration was stopped by heating the reaction mixture to 150 °C for approximately 2 h where the catalyst is decomposed. Cyclic side products, were removed at 150 °C at a vacuum of 0.1 mbar for about 5 h using a Schlenk-Line. Yield: 57.35 g; 82%. Titrated ${\bar{M}}_{n}$: 3280 g mol^−^^1^; ${\bar{M}}_{n}$ from ^1^H NMR: 3416 g mol^−1^.

### 2.2 Determination of molecular weight

#### 2.2.1 Theoretical number average molecular weight

Values for the theoretical ${\bar{M}}_{n}$ of the polysiloxanes were calculated from the initial weights of the monomers and from the conversion. (See Eq. (1) in research article [1]).

#### 2.2.2 Titration

Titration of the amino end-groups was also used to determine the number average molecular weight of the polysiloxanes. 1.5–1.7 g of the polysiloxanes were dissolved in 50 mL THF and were titrated with 0.1 M HCl using bromophenol blue until a color change from blue to yellow was observed. The molecular weights were calculated from an average of 3 titrations and the mean values were used for the calculation of the reaction stoichiometry of the subsequent synthesis of polysiloxane-urea-elastomers.

#### 2.2.3 NMR spectroscopy

**Fig. 2:**
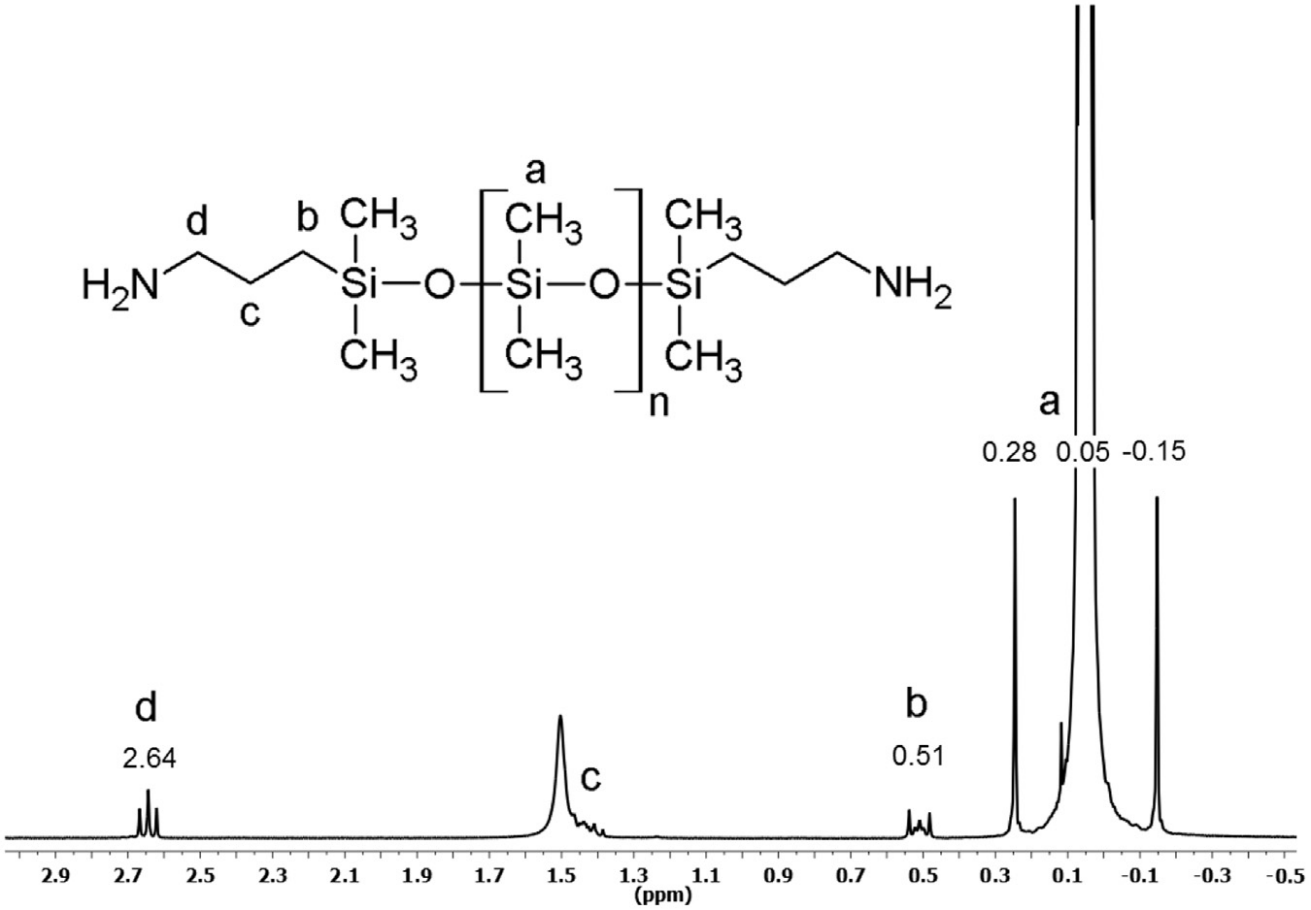
^1^H NMR spectrum of an α,ω-Bis(3-aminopropyl)-polydimethylsiloxane. For calculation of the molecular weight, the integral values of the methylene protons d (δ 2.64 ppm) and b (δ 0.51 ppm) and methyl protons a (around δ 0.05 ppm) were used. The signal c (around δ 1.5 ppm) is overlaid by a HDO signal and was therefore not used for calculation of the molecular weight.

^1^H NMR spectra were used to determine the number average molecular weight ${\bar{M}}_{n}$ of the polydimethylsiloxanes and polydimethyl-methyl-phenyl-siloxane-copolymers. About 10–20 mg of the polysiloxanes were dissolved in 0.5 mL CDCl_3_. Chemical shifts [δ] were calibrated to the CDCl_3_ solvent peak at 7.26 ppm. ^29^Si NMR spectra were used to evaluate the amount of incorporated methyl-phenyl-siloxane within the PDMS-Me,Ph-copolymers. Approximately 150 mg of the PDMS-Me,Ph-copolymers were dissolved in CDCl_3_ and 50 mg of the relaxation agent Chromium(III)-acetylacetonate was added to the samples. Fig. 2 shows a ^1^H NMR spectrum of an aminopropyl-terminated polydimethylsiloxane. The signals of the methylene protons *b, c* and *d* within the two propyl-chains can be clearly distinguished from the broad sum signal *a* of the methyl protons from the dimethylsiloxane-repeating unit. The signal at around *δ* 1.5 ppm, however, is overlaid by a broader signal of residual water traces, which undergoes proton exchange with the solvent CDCl_3_ to form HDO [3]. Therefore, the integral of this signal cannot be used for calculation of the molecular weight.

**Fig. 3:**
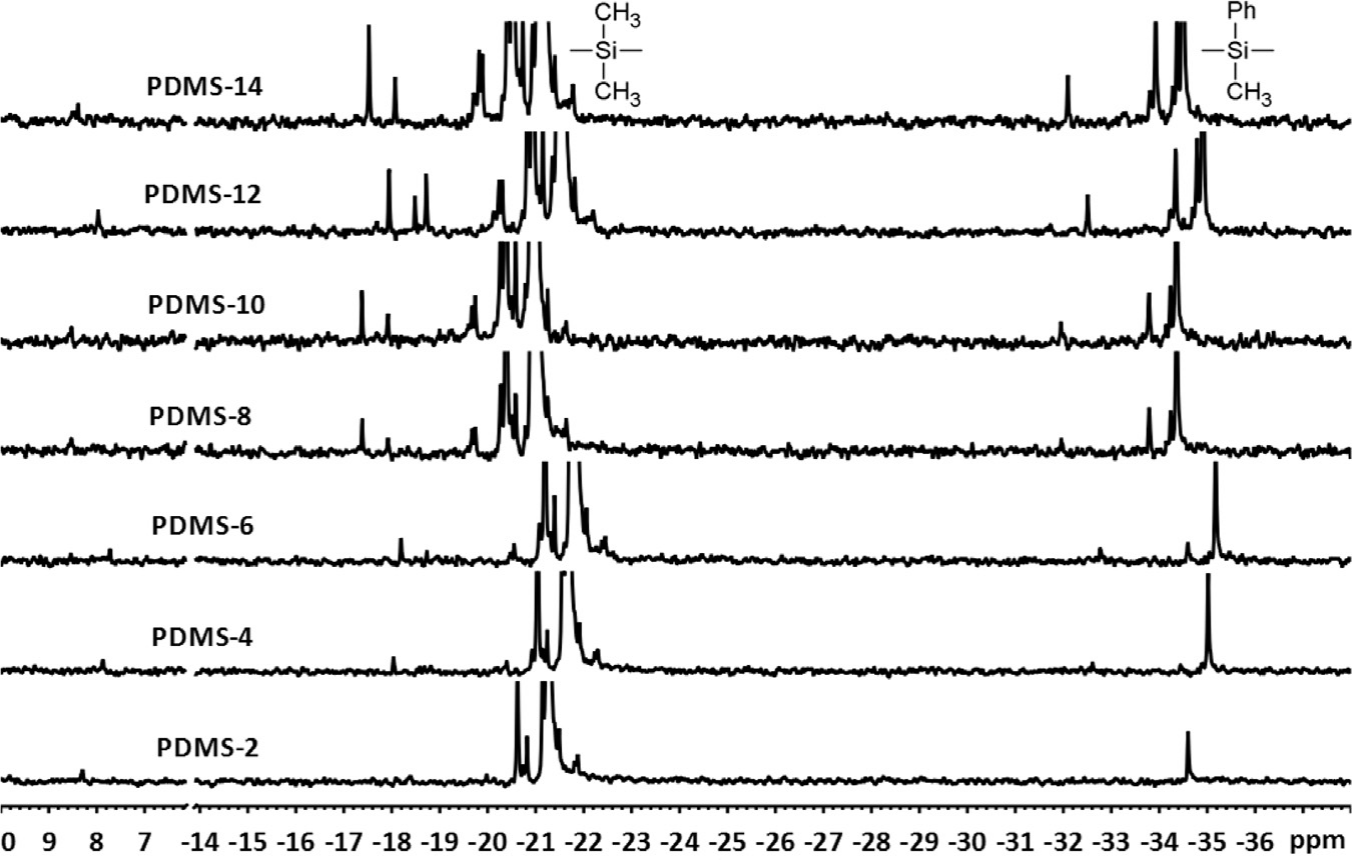
^29^Si NMR spectra of synthesized α,ω-Bis(3-aminopropyl)-polydimethyl-methyl-phenylsiloxane-copolymers with different amounts of incorporated methyl-phenyl-siloxane-units ranging from 2 to 14 mol%.

Fig. 3 shows a series of ^29^Si NMR spectra of α,ω-Bis(3-aminopropyl)-polydimethyl-methyl-phenylsiloxane-copolymers. The numbers represent the amount of incorporated methyl-phenyl-siloxane (mol%) within the copolymer. The small signal around *δ* 8 ppm is attributed to the terminal dimethyl-siloxane-units. The signals between *δ* −20 and −22 ppm are assigned to the dimethyl-siloxane repeating units. The signal(s) for the methyl-phenyl-siloxane-units appear(s) between *δ* −32 and −35 ppm. The signal intensity of the methyl-phenyl-siloxane-units not only increases, there is also a signal splitting with increasing amounts of methyl-phenyl-siloxane. This appears as a second signal around *δ* −32 ppm, which can be attributed to a triad of methyl-phenyl-siloxane-units. The larger signals between *δ* −34 and −35 ppm represent a methyl-phenyl-siloxane-unit, which is adjacent to one another and to a dimethyl-siloxane-unit. A similar signal splitting is apparent for the silicon atoms within a dimethyl-siloxane-repeating unit [4], [5]. It can therefore be presumed that larger sequences of adjacent methyl-phenyl-siloxane-units were incorporated into the PDMS-chain with increasing concentrations of D_4_^Me,Ph^.

#### 2.2.4 Size exclusion chromatography

SEC measurements were performed on polysiloxane-urea-elastomers to determine the number and weight average molecular weights and their corresponding polydispersity indices (PDIs) (see Table 3, Table 4). PSU-solutions (in THF) were measured at 40 °C with a flow rate of 0.5 mL/min. Molecular weights were calibrated with polystyrene standards.

**Table 3:** *M* ratios and molecular weights (obtained by SEC) of prepared polydimethylsiloxane-urea elastomers. The polymer notation refers to the molecular weight of the PDMS, used for synthesis of the PSU-elastomers. (PSU-3T=PSU with PDMS molecular weight of 3000 g mol^−1^).

**PSU**	**Molar ratio (PDMS/H**_**12**_**MDI/APTMDS)**	${\bar{M}}_{n}$**SEC**	${\bar{M}}_{w}$**SEC**	**PDI**
**PSU-3T**	1.0/1.2/0.2	135,700	169,200	1.25
**PSU-6T**	1.0/1.8/0.8	129,800	181,300	1.40
**PSU-9T**	1.0/2.6/1.6	155,000	185,700	1.20
**PSU-12T**	1.0/3.2/2.2	143,400	170,700	1.19
**PSU-15T**	1.0/3.7/2.7	144,000	190,300	1.32
**PSU-18T**	1.0/4.5/3.5	131,100	165,600	1.26
**PSU-23T**	1.0/5.4/4.4	131,500	164,200	1.25
**PSU-26T**	1.0/6.1/5.1	158,200	185,400	1.17
**PSU-31T**	1.0/7.2/6.2	125,300	160,900	1.28
**PSU-33T**	1.0/7.6/6.6	126,500	161,700	1.28

**Table 4:** *M* ratios and molecular weights (obtained by SEC) of prepared polydimethylsiloxane- methyl-phenyl-siloxane-copolymers. The polymer notation refers to the to the incorporated amount of methyl-phenyl-siloxane (mol%) of the PDMS, used for synthesis of the PSU-elastomers.

**Polymer**	**molar ratio (PDMS/H**_**12**_**MDI/APTMDS)**	${\bar{M}}_{n}$**SEC**	${\bar{M}}_{w}$**SEC**	**PDI**
**PSU-Ph2**	1.0/4.5/3.5	132,800	173,900	1.31
**PSU-Ph4**	1.0/4.5/3.5	143,600	177,100	1.23
**PSU-Ph6**	1.0/4.9/3.9	122,800	164,900	1.34
**PSU-Ph8**	1.0/4.8/3.8	127,800	170,900	1.34
**PSU-Ph10**	1.0/4.7/3.7	138,500	184,000	1.33
**PSU-Ph12**	1.0/5.0/4.0	113,000	155,200	1.37
**PSU-Ph14**	1.0/4.8/3.8	122,900	167,600	1.36

### 2.3 Characterization of polysiloxane-urea synthesis by FTIR spectroscopy

#### 2.3.1 *In line* FTIR-ATR spectroscopy

*In line* FTIR-ATR spectroscopy was applied in one PSU-synthesis^1^ in order to monitor the reaction progress of isocyanate (H_12_MDI) conversion with α,ω-Bis(3-aminopropyl)-polydimethylsiloxane and APTMDS. Spectra were recorded using a Mettler Toledo ReactIR 45 m^®^ ATR-FTIR spectrometer equipped with a SiComp (Silicon) probe connected to the spectrometer via a silver halide fiber (9.5 mm×2 m). Spectra within a range of 2500 and 650 cm^−1^ were recorded every 15 s with a resolution of 4 cm^−1^ using Mettler Toledo iC IR^®^ software version 4.3.35 SP1.

The reaction procedure was as follows: In a 250 mL four-neck, round-bottom reaction flask equipped with a PTFE oval-shaped magnetic stir bar, dropping funnel, nitrogen in- and outlet and the inserted ATR-probe, the desired amount of H_12_MDI was dissolved in THF. The spectra collection was started to record the initial NCO-concentration by following the height of the NCO absorption peak at 2263 cm^−1^. 31.5 g of undiluted PDMS was then added quickly (within 38 s) to the H_12_MDI-solution through the dropping funnel. After the NCO peak height remained constant again, approximately 50 mL of THF, used to rinse the dropping funnel, were also added to the prepolymer-solution. Finally, the total amount of the chain extender APTMDS (calculated according to the reaction stoichiometry) was added quickly via a syringe. PSU-formation was indicated by an instantaneous increase of viscosity. The PSU-solution was therefore diluted with THF to a final concentration of 17% (w/w).

**Fig. 4:**
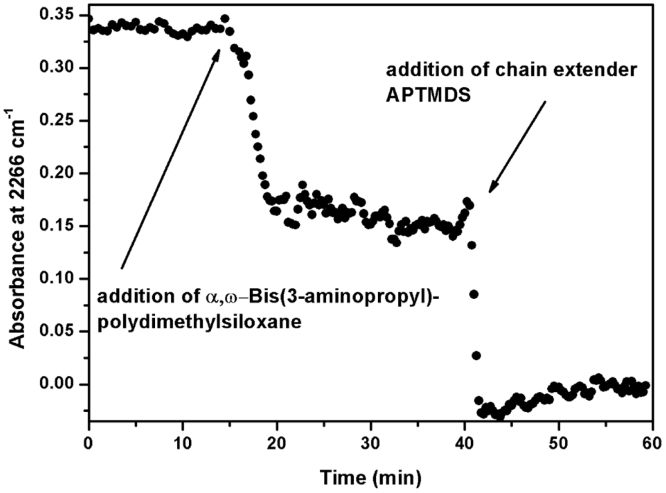
Reaction progress of polydimethylsiloxane-urea (PSU) synthesis followed by in line FTIR-ATR spectroscopy. The peak height of the NCO-absorption at 2266 cm^−^^1^ was used to follow the conversion of isocyanate groups. Immediately after addition of α,ω-Bis(3-aminopropyl)-polydimethylsiloxane, the NCO peak decreased, indicating formation of NCO-terminated prepolymer-chains. After addition of the chain extender APTMDS, the NCO peak disappeared completely from the IR spectra.

**Fig. 5:**
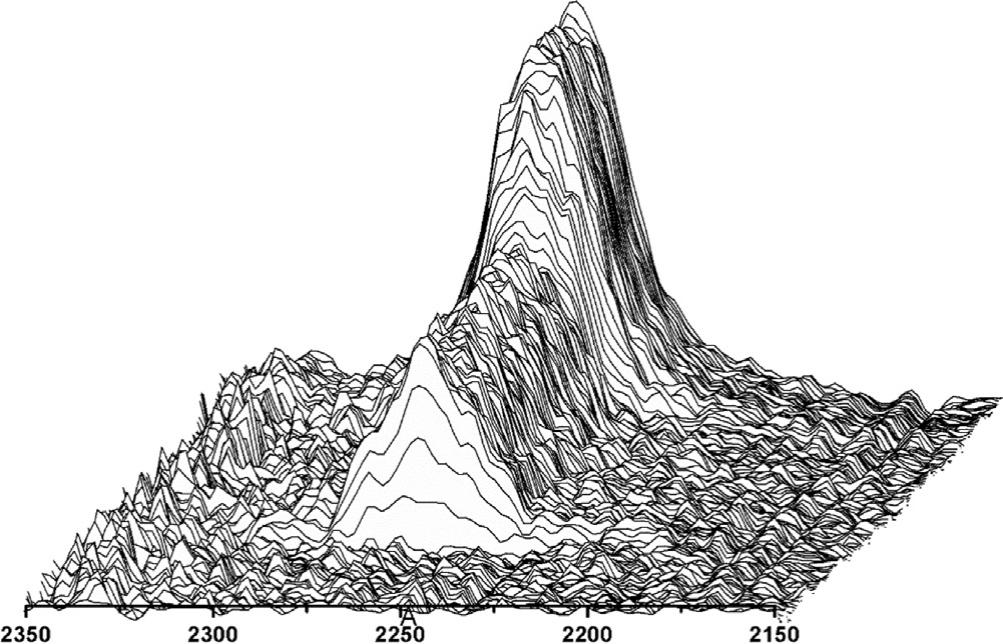
Time-dependent plot of the NCO-absorption peak followed by in line FTIR-ATR spectroscopy during synthesis of polydimethylsiloxane-urea (PSU).

The reaction profile for the synthesis of a polydimethylsiloxane-urea is shown in Fig. 4 and Fig. 5. For improved visualization, the following graphs were created using smoothed (moving-average) spectral data. Fig. 4, Fig. 5 show that the NCO peak height decreased immediately after addition of the amino-terminated PDMS, which indicated the formation of NCO-terminated prepolymer-chains. After the NCO peak height remained constant again, a small portion of THF was added to the prepolymer-solution, leading to a negligible decrease of the NCO peak height, through a dilution effect (after 30 min reaction time). The chain-extension-step proceeded very fast, as indicated by the steep decline and final disappearance of the NCO peak.

#### 2.3.2 *Off line* FTIR-ATR spectroscopy

*Off line* FTIR-ATR spectra were recorded on a Perkin Elmer FTIR spectrometer (Frontier^®^) equipped with a ZnSe-Diamond ATR unit using Spectrum^®^ software version 10.4.3.

IR spectra are given as an average of 6 scans with a resolution of 2 cm^−^^1^.

**Fig. 6:**
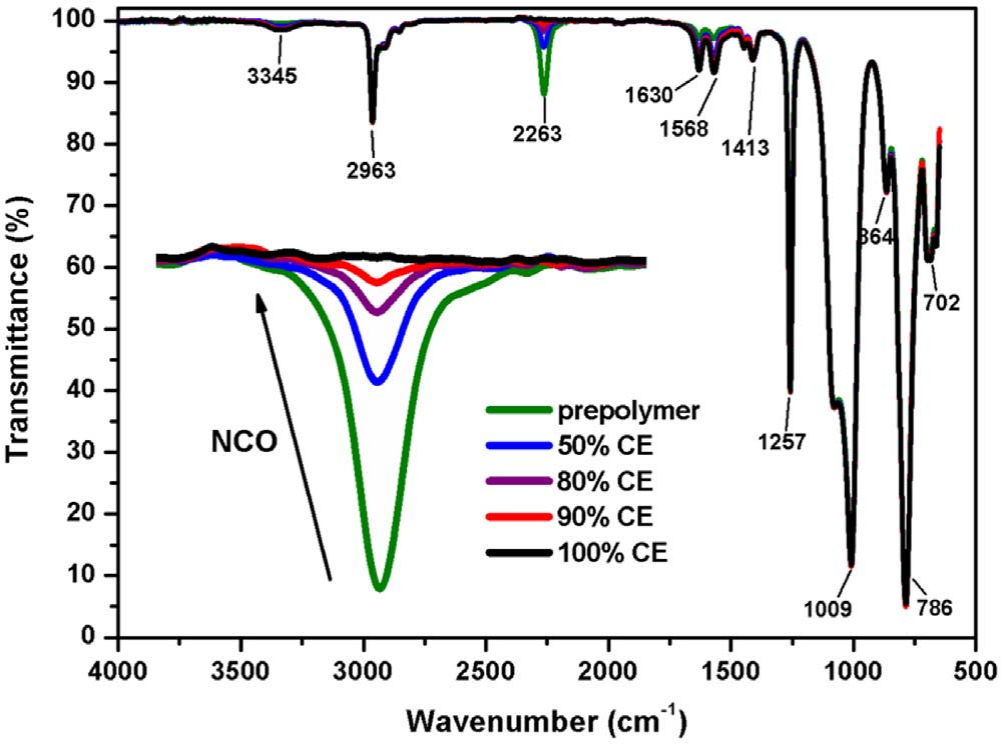
Synthesis of a polydimethylsiloxane-urea-elastomer, followed by ATR-FTIR spectroscopy. Reaction progress is indicated by the step-wise reduction of the NCO absorption peak at 2263 cm^−^^1^. After formation of the prepolymer (green), portions of the chain extender (CE) APTMDS were added according to calculated stoichiometry until complete disappearance of the NCO peak.

During synthesis, samples (in THF) were taken at different times (after prepolymer formation and after each addition of the chain extender (CE)) to monitor reaction progress of the polyaddition. IR-spectra were measured from thin polymer films, which were produced at the ATR crystal by evaporation of the solvent in a continuous nitrogen flow. The synthesis of polysiloxane-urea was completed after the NCO absorption peak at 2263 cm^−1^ disappeared completely from the IR spectrum, as indicated by the arrow in Fig. 6. Table 5 gives the band assignments in the IR spectrum of a polydimethylsiloxane-urea.

**Table 5:** Band assignments in FTIR spectra of polydimethylsiloxane-ureas [6], [7].

**Wavenumber (cm**^**−1**^**)**	**Assignment**
3345	υ (N-H) hydrogen-bonded; urea
2963	υ_as_ (C-H); CH_3_
2263	υ_as_ (N=C=O)
1630	υ (C=O) hydrogen-bonded; Amide I stretch; urea
1658	υ (C-N) + δ (N-H); Amide II stretch & bend; urea
1413	δ_s_ (C-H); CH_3_
1257	δ_s_ (C-H); CH_3_
1009	υ_as_ (Si-O-Si)
864	δ_as_ (C-H) rocking; Si(CH_3_)_2_
786	υ_as_ (Si-C); Si(CH_3_)_2_
702	υ_s_ (Si-C); Si(CH_3_)_2_

### 2.4 Mechanical characterization of polysiloxane-urea-elastomers

Polymer sheets (0.30–0.45 mm) were prepared by casting of polymer solutions into glass Petri dishes. The solvent CHCl_3_ was slowly evaporated at room temperature overnight by covering the Petri dishes with a perforated aluminium foil. Petri dishes were placed under the fume-hood with the sash window left open. Dog-bone shaped test specimens (DIN EN 53504, type S2) were die cut from these sheets. Stress-strain measurements were performed by stretching the specimens having an original length (L_0_) of 20 mm until break with a crosshead speed of 25 mm/min. A pre-load of 0.1 MPa was applied. The values for Young's Modulus, Ultimate Tensile Strength and Elongation at Break (see Table 6) were calculated as a mean of 5 repeated measurements.

**Table 6:** Young's modulus, ultimate tensile strength and elongation at break of polydimethylsiloxane-based urea-elastomers. The polymer notation refers to the molecular weight of the PDMS, used for synthesis of the PSU-elastomers. (PSU-3T=PSU with PDMS molecular weight of 3000 g mol^−^^1^).

**PSU**	**Young's modulus (MPa)**	**Ultimate tensile strength (MPa)**	**Elongation at break (%)**
**PSU-3T**	5.52±0.36	6.05±1.03	880±59
**PSU-6T**	3.66±0.24	5.22±0.43	877±101
**PSU-9T**	1.78±0.10	4.47±1.07	899±189
**PSU-12T**	1.44±0.17	4.14±0.77	796±101
**PSU-15T**	1.03±0.07	3.83±0.38	1280±84
**PSU-18T**	0.99±0.13	3.48±0.85	717±159
**PSU-23T**	0.74±0.03	2.16±0.34	504±105
**PSU-26T**	0.61±0.02	1.91±0.21	593±84
**PSU-31T**	0.57±0.12	1.91±0.45	533±94
**PSU-33T**	0.59±0.02	1.31±0.09	358±38

10-cycle hysteresis measurements were performed with a crosshead speed of 25 mm/min until an elongation of 100% was reached. Specimens were immediately released with the same crosshead speed and the consecutive cycles were started after the crosshead returned to the initial starting position. Values for mechanical hysteresis after each cycle were obtained by calculating the areas of the corresponding loading and unloading curves and are displayed in Table 7 as mean of 3 repeated measurements.

**Table 7:** Mechanical hysteresis at 100% elongation of polydimethylsiloxane-based urea-elastomers.

**PSU /cycle**	**1**	**2**	**3**	**4**	**5**	**6**	**7**	**8**	**9**	**10**
**3T**	54	39	36	35	34	33	33	32	32	32
**SD**	0.4	0.2	0.1	0.1	0.1	0.0	0.0	0.0	0.0	0.4
**6T**	43	27	24	23	23	22	22	21	21	21
**SD**	0.3	0.6	0.4	0.2	0.2	0.1	0.1	0.0	0.1	0.0
**9T**	18	12	10	10	10	9	9	9	9	9
**SD**	2.6	0.5	0.3	0.2	0.2	0.2	0.2	0.2	0.0	0.0
**12T**	20	7	6	6	5	5	5	5	5	5
**SD**	1.1	0.4	0.7	0.8	0.8	0.9	0.8	0.9	0.9	0.9
**15T**	14	7	6	6	6	5	5	5	5	5
**SD**	2.1	0.6	0.3	0.3	0.3	0.3	0.2	0.2	0.3	0.2
**18T**	9	5	4	4	4	4	4	4	4	4
**SD**	0.3	0.3	0.1	0.1	0.0	0.1	0.2	0.2	0.4	0.3
**23T**	12	6	5	5	5	5	5	5	5	4
**SD**	0.8	0.8	0.8	0.8	0.8	0.8	0.7	0.7	0.8	0.7
**26T**	8	2	2	2	2	2	2	2	2	2
**SD**	0.5	0.2	0.2	0.1	0.2	0.1	0.2	0.2	0.2	0.2
**31T**	9	3	3	3	3	3	3	3	3	3
**SD**	4.3	1.0	1.1	1.1	1.0	1.0	1.0	1.0	1.0	1.0
**33T**	6	3	2	2	2	2	2	2	2	2
**SD**	0.4	0.1	0.0	0.0	0.1	0.1	0.1	0.1	0.1	0.1

## Footnotes

1 For reasons of comparability, this PSU was not involved in optical and mechanical characterization, described in the related research article [1] because polyaddition proceeded mainly with undiluted reactants.
