# Supplementary material for: Data on the synthesis and mechanical characterization of polysiloxane-based urea-elastomers prepared from amino-terminated polydimethylsiloxanes and polydimethyl-methyl-phenyl-siloxane-copolymers
Source: Data Brief. 2018 Apr 30;18:1784–94. doi: 10.1016/j.dib.2018.04.083 (PMC5998215; doi:10.1016/j.dib.2018.04.083)
Supplement: Supplementary file 1 — Supplementary material [file mmc1.docx]

**Conflict of Interest**

The authors of the manuscript entitled **“Data on the synthesis and mechanical characterization of polysiloxane-based urea-elastomers prepared from amino-terminated polydimethylsiloxanes and polydimethyl-methyl-phenyl-Sloane-copolymers”** declare no conflict of interest.
